# A Tripeptide (Ser-Arg-Pro, SRP) from *Sipunculus nudus* L. Improves Cadmium-Induced Acute Kidney Injury by Targeting the MAPK, Inflammatory, and Apoptosis Pathways in Mice

**DOI:** 10.3390/md22060286

**Published:** 2024-06-20

**Authors:** Yanmei Pan, Zhilan Peng, Zhijia Fang, Lukman Iddrisu, Lijun Sun, Qi Deng, Ravi Gooneratne

**Affiliations:** 1 Guangdong Provincial Key Laboratory of Aquatic Product Processing and Safety, Guangdong Provincial Engineering Technology Research Center of Marine Food, Key Laboratory of Advanced Processing of Aquatic Products of Guangdong Higher Education Institution, College of Food Science and Technology, Guangdong Ocean University, Zhanjiang 524088, China; 2112103106@stu.gdou.edu.cn (Y.P.); lukmaniddrisu54@gmail.com (L.I.); suncamt@126.com (L.S.); gdoudengqi@163.com (Q.D.); 2 School of Ocean and Tropical Medicine, Guangdong Medical University, Zhanjiang 524023, China; pengzhilan@gdmu.edu.cn; 3 Department of Wine, Food and Molecular Biosciences, Lincoln University, Lincoln 7647, New Zealand; ravi.gooneratne2@lincolnuni.ac.nz

**Keywords:** cadmium-induced kidney injury, SRP, network pharmacology, oxidative stress, apoptosis regulation

## Abstract

Cadmium (Cd) is a toxic heavy metal that causes nephrosis, including acute kidney injury. To prevent and treat acute kidney injury (AKI) following Cd exposure, a tripeptide, Ser-Arg-Pro (SRP), from *Sipunculus nudus* L. was employed, and its potential efficacy in AKI was assessed. Oral administration of SRP significantly alleviated Cd-induced kidney damage, leading to improved renal function and the attenuation of structural abnormalities. A network pharmacology analysis revealed the potential of SRP in renal protection by targeting various pathways, including mitogen-activated protein kinase (MAPK) signaling, inflammatory response, and apoptosis pathways. Mechanistic studies indicated that SRP achieves renal protection by inhibiting the activation of MAPK pathways (phosphorylation of p38, p56, ERK, and JNK) in the oxidative stress cascade, suppressing inflammatory responses (iNOS, Arg1, Cox2, TNF-α, IL-1β, and IL-6), and restoring altered apoptosis factors (caspase-9, caspase-3, Bax, and Bcl-2). Hence, SRP has the potential to be used as a therapeutic agent for the treatment of Cd-induced nephrotoxicity.

## 1. Introduction

Cadmium (Cd) is a highly prevalent heavy metal pollutant that poses a significant hazard to human health. It has the potential to induce severe toxic effects in many organs, such as the kidneys and liver [1]. Epidemiological data show that exposure to Cd causes severe damage to many organs, including nephrotoxicity, hepatotoxicity, and pneumotoxicity [2,3]. Exposure to low levels of Cd can cause damage to the skeletal, hemopoietic, and cardiovascular systems, as well as impaired vision and hearing. Moreover, strong teratogenic and mutagenic effects have been associated with cadmium, with low doses exerting adverse effects on both male and female reproduction and affecting pregnancy or its outcome [4,5]. In addition, Cd and its compounds were classified as type 1 carcinogens by the International Agency for Research on Cancer in 1993 [6]. Among the affected organs, the kidneys are highly vulnerable to Cd exposure, with approximately half of the accumulated Cd residing in the kidneys, particularly within the proximal convoluted tubules, leading to renal dysfunction and the development of chronic kidney disease [7]. Acute kidney injury (AKI) refers to a rapid decline in renal function over a short period [8]. Numerous studies have demonstrated that Cd exposure can trigger the onset of AKI, with endogenous reactive substances and pathways playing pivotal roles [9,10]. Approximately 50% of the Cd accumulates in the kidney, particularly in the proximal convoluted tubules, leading to renal dysfunction and chronic kidney disorders [11]. Cd exposure triggers nephrotoxicity through mechanisms involving inflammation, apoptosis, and the generation of reactive oxygen species (ROS) [12,13]. Previous studies have suggested that up to 7 percent of the global population suffers from chronic kidney disease due to Cd exposure [14]. Finding a way to effectively mitigate Cd-induced AKI has always been a challenge.

Currently, the chelation therapy administered for Cd toxicity is not fully effective for removing Cd from the body [15]. However, since the developed synthetic treatment agents have adverse effects, the mitigation of tissue damage caused by Cd toxicity with natural antioxidants has come to the foreground [16].

The peanut worm, also known as *Sipunculus nudus* Linnaeus, is a marine organism with diverse pharmacological activities and has garnered attention for its antioxidant, radioprotective, antibacterial, anti-fatigue, and immunomodulatory properties [17,18,19]. Peanut worms, rich in essential amino acids and polysaccharides, exhibit anti-fatigue benefits, inhibit blood clot formation, and exhibit anti-inflammatory properties [20]. The hydrolysate peptides from *S. nudus* L. exhibited remarkable scavenging activities against superoxide anions, DPPH radicals, and hydroxyl radicals, along with notable ACE inhibitory activity and in vitro antitumor properties [21]. Studies have shown that amino acids, such as Ser, Arg, and Pro, can improve renal dysfunction and reduce renal disease [22,23,24]. Peptides rich in Ser, Arg, and Pro exhibit good anti-Cd and nephroprotective effects [25,26,27,28].

In this study, for the prevention and treatment of Cd-induced AKI, a biologically active tripeptide, Ser-Arg-Pro (SRP) was isolated from the hydrolysis of *S. nudus* L. [29]. There is no clear information in the literature on whether SRP has a protective effect against kidney damage caused by Cd. However, a large number of studies have shown that kidney injury caused by cadmium is closely related to oxidation, inflammation, and apoptosis, which are also functional activities of SRP. Therefore, the purpose of this study is to investigate the protective effects of SRP on oxidative stress, inflammation, and apoptosis pathways in Cd-induced kidney injury using molecular, biochemical, and histopathological methods.

## 2. Results and Discussion

### 2.1. Effects of SRP on Body Weight and Organ Indices

**Figure 1:**
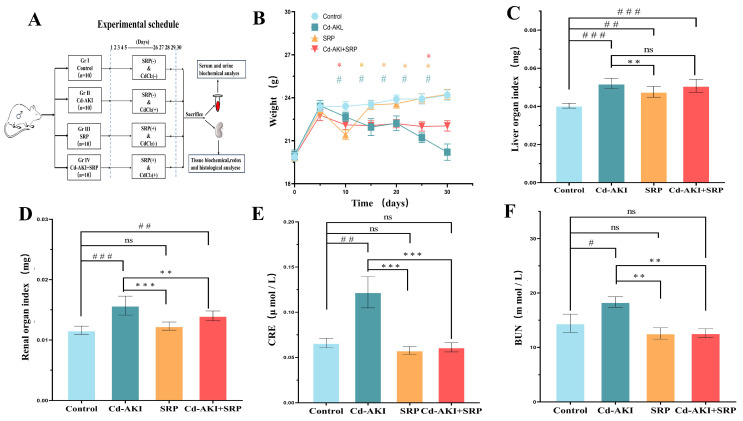
Effect of SRP on general body condition in mice. (**A**) A schematic of the in vivo experimental protocol. (**B**) Weight. (**C**) Liver organ index. (**D**) Renal organ index. (**E**) BUN. (**F**) CRE. “^#^” indicates values that significantly (*p* < 0.05) differ from control. “^##^” indicates values that significantly differ from control (*p* < 0.01). “^###^” indicates values that significantly differ from control (*p* < 0.001). “*” indicates values that significantly differ from Cd-AKI (*p* < 0.05). “**” indicates values that significantly differ from Cd-AKI (*p* < 0.01). “***” indicates values that significantly differ from Cd-AKI (*p* < 0.001). “ns” indicates no significant difference.

The experimental design is illustrated in Figure 1A. Regular monitoring of the body weight revealed a consistent decline in the Cd group. Notably, the SRP group exhibited a discernible increase in body weight after a 20-day treatment compared with the Cd-AKI group (Figure 1B).

The kidney-to-body weight ratio (Kw/Bw) is an indicator of kidney health. Cd treatment significantly elevated the Kw/Bw ratio compared with that of the normal control, indicating compromised kidney health. In contrast, SRP treatment resulted in a significant reduction (*p* < 0.05) in the Kw/Bw ratio compared with that of the Cd-AKI group. Additionally, SRP treatment showed a non-significant reduction in the ratio compared to that of the control group (Figure 1B). The trends observed in the liver index were consistent with those observed in the kidney index (Figure 1C,D). Body weight reflects an animal’s health status and absorption of nutrients, and a decline in the health status of mice is reflected by weight loss. Consistent with the results of previous [30] studies, the changes in mouse body weight in this experiment showed that the heavy metal Cd hindered the growth of mice, while SRP alleviated the toxicity of Cd in mice. The growth inhibition in mice may be due to insufficient digestion and absorption of nutrients in the intestinal mucosa caused by Cd. The kidney coefficient is a necessary test item in toxicology research. Increased organ coefficients indicate organ congestion and edema, while decreased organ coefficients indicate organ atrophy and other degenerative changes. Studies have shown that long-term exposure to 10 mg/kg Cd increases liver and spleen weights [31]. Here, we observed that the kidney coefficients were increased by Cd, and that SRP antagonized the increase in kidney weight caused by Cd.

Biomarkers of Cd-induced AKI, including blood urea nitrogen (BUN) and creatinine (CRE), reflect renal filtration function and increase in the blood as renal function declines. However, SRP treatment significantly decreased the BUN and CRE levels, indicating its potential efficacy in mitigating Cd-induced renal pathological damage caused by Cd toxicity (Figure 1E,F). Previous studies have reported that peptide support can effectively reduce elevated CRE levels [32]. Protein support could also significantly attenuate BUN level and improve proteinuria in patients with nephropathy [33,34]. Previous studies have also reported that Ser and Arg alone do not significantly reduce BUN or CRE levels [35,36]. These results suggest that SRP may be promising in alleviating adverse renal effects induced by Cd exposure.

### 2.2. Histological Analysis

**Figure 2:**
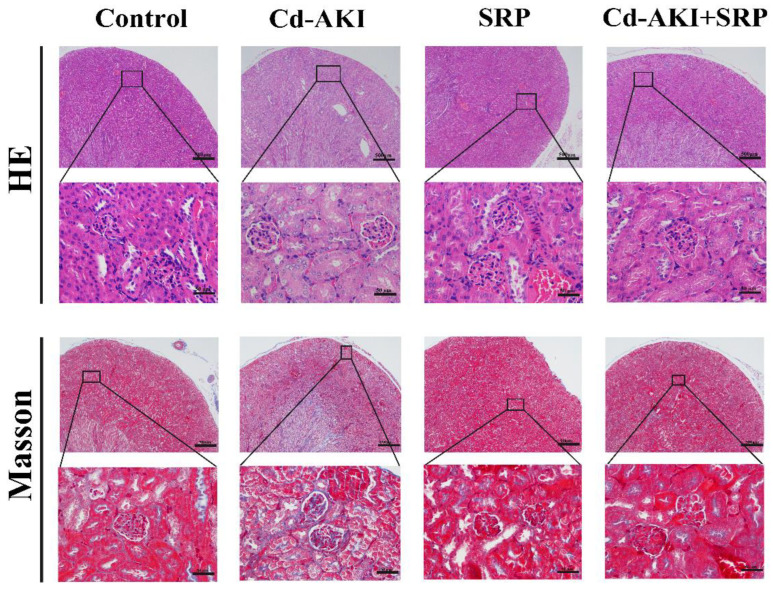
Histological assessments of kidneys of experimental mice in the absence (CdCl_2_) and presence of SRP (CdCl_2_ + SRP). Bar = 500/50 µm.

The histological assessment, illustrated in Figure 2, revealed distinctive features of renal tissue morphology. In the control group, H&E staining revealed intact and well-organized glomerular and tubular structures with clear lumens and evenly stained proximal convoluted tubular epithelial cells. Conversely, mice exposed to Cd displayed severe histopathological alterations including renal glomerular atrophy, swollen proximal convoluted tubule epithelial cells, and tubular degeneration and dilation. SRP treatment significantly alleviated these histopathological changes, ameliorating the overall pathohistological outcomes in injured kidney tissues. Previous studies have shown that Arg effectively reduces gentamicin-induced acute tubular necrosis [37]. The Arg-Gly-Asp peptide enhanced the therapeutic efficacy of extracellular vesicles in renal repair and decreased tubular injury in AKI mice [38]. Peptides containing Pro also showed a good alleviative effect on tubular epithelial apoptosis [39].

Masson staining of renal sections revealed a substantial increase in collagen deposition in renal tissue following Cd-induced AKI. Intriguingly, SRP treatment in the Cd-AKI group markedly reduced renal tissue collagen deposition. It was noteworthy that the oral administration of SRP alone at a dose of 50 mg/kg did not elicit discernible effects on renal tissue structure, glomerular matrix dilation, or renal collagen deposition. Collagen is a necessary structural framework for renal tissue [40]. Previous studies have reported that orally administered peptides can promote pro-collagen synthesis and inhibit collagen degradation [41]. Pro is required to form collagen molecules and to regulate collagen biosynthesis [42]. These findings underscore the potential of SRP in mitigating renal morphological alterations and collagen deposition induced by Cd exposure.

These histomorphological changes may reflect a direct impairment of the renal tissues. Our findings are further supported by those of Kamel et al. [43], who reported that Cd administration led to histomorphological changes in renal tissues, such as tubular and glomerular atrophy. It is speculated that the histomorphological changes may be due to the excessive production of ROS caused by Cd exposure [44], which results in oxidative impairment [45] and morphological changes in the renal tissue. However, the Cd-AKI + SRP group displayed mild to moderate vacuolation in the tubular epithelium. Furthermore, the co-administration of Cd and SRP significantly mitigated the above-mentioned histopathological damage. This may be attributed to the antioxidant capability of SRP, which significantly decreases oxidative stress, leading to a decrease in pathological alterations.

### 2.3. Biochemical Markers of Kidney Function

**Figure 3:**
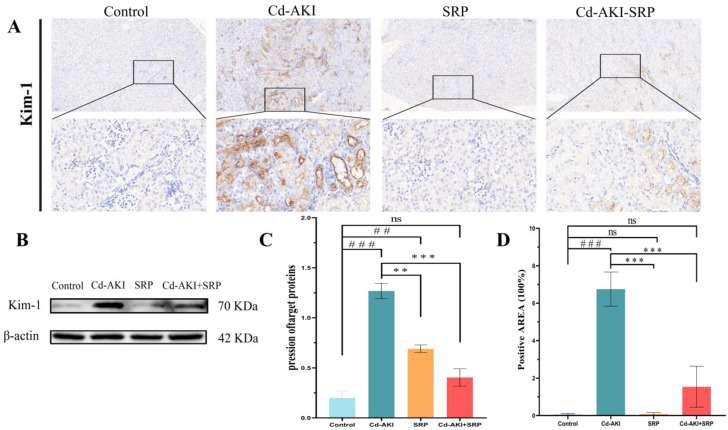
SRP reduces Kim-1 expression, a marker of renal damage, in Cd-AKI mouse kidneys. (**A**) Immunohistochemical staining of Kim-1 in the kidneys of Cd-AKI mice. Bar = 100/20 µm. (**B**) Relative expression of Kim-1. (**C**) Quantification of Kim-1 protein expression density. (**D**) Immunohistochemical density determination of Kim-1 expression. All data are presented as the mean ± SD. “^##^” indicates values that significantly differ from the control group (*p* < 0.01). “^###^” indicates values that significantly differ from control (*p* < 0.001). “**” indicates vales that are significantly different from Cd-AKI (*p* < 0.01). “***” indicates values that significantly differ from Cd-AKI (*p* < 0.001). “ns” indicates no significant difference.

Kim-1 serves as an early marker of tubular injury and guides renal protective interventions [46]. A significant upregulation of Kim-1 was observed in Cd-induced renal damage [47]. In this study, we evaluated the effect of SRP on the expression of Kidney Injury Molecule-1 (Kim-1) in AKI mice using Western blotting (WB) and immunohistochemical (IHC) assays (Figure 3). This study revealed a significant upregulation of Kim-1 expression due to Cd exposure. In contrast, oral administration of SRP led to a notable reduction in Kim-1 expression. Furthermore, the IHC data indicated that the combined treatment of SRP and Cd resulted in diminished positive staining of Kim-1 compared to that in mice treated with Cd alone. These results illustrate that SRP improves renal dysfunction in Cd-induced AKI in mice. Earlier studies have shown that AKI upregulates Kim-1 expression through various pathways, including the inflammatory response and apoptosis [48]. However, the role of SRP in mitigating Cd-induced AKI requires further investigation.

### 2.4. Exploration of Potential Targets and Networks of SRP

**Figure 4:**
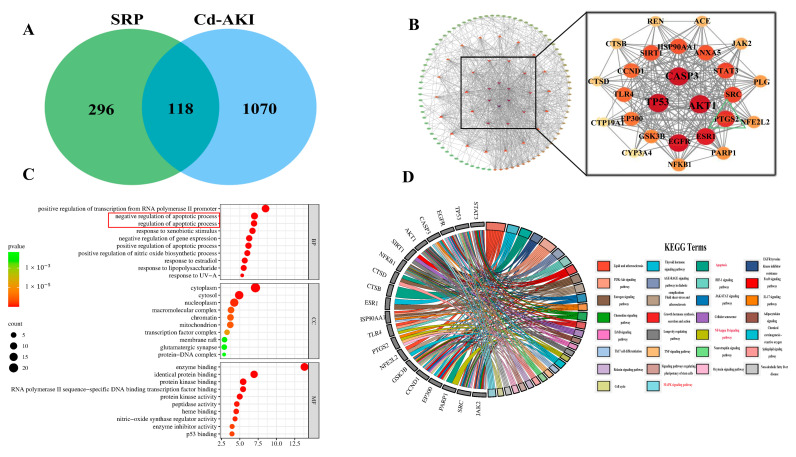
Pharmacological analysis of the SRP–AKI network. (**A**) Venn diagram illustrating overlapping target genes. (**B**) Protein–protein interaction (PPI) network. (**C**) Kyoto Encyclopedia of Genes and Genomes (KEGG) enrichment analysis results. (**D**) Gene Ontology (GO) enrichment analysis results.

Given the encouraging efficacy of SRP in addressing Cd-induced AKI, we aimed to understand the mechanisms underlying its efficacy. We adopted a treatment approach based on a network pharmacological analysis. Initially, 188 pathogenic genes/targets associated with Cd-induced acute infection were identified in the database (Figure 4A). Simultaneously, we identified 414 pharmacological genes/targets associated with SRP using database screening. The Venn diagram analysis revealed a significant overlap of 118 genes/targets between the pharmacological profile of SRP and the genetic basis of Cd-induced acute kidney injury, ultimately identifying 118 potential targets associated with SRP for the treatment of Cd-induced AKI. Protein–protein interactions (PPIs) were subsequently constructed from these targets (Figure 4B) and the top 25 key genes were identified based on their degree values. These key genes may play important roles in the treatment of Cd-induced AKI with SRP.

To delve deeper into the functional relevance of these targets, we conducted functional and pathway enrichment analyses of the 25 core targets. Using the DAVID database, we performed a comprehensive Gene Ontology (GO) functional analysis. To provide a concise summary, we selected the top 10 entries from each classification, as presented in Figure 4C.

Additionally, a KEGG pathway analysis was performed on the 25 core targets, revealing significant enrichment across 102 signaling pathways. The top 30 pathways are shown in Figure 4D. These pathways do not include various areas associated with viral infections and cancer, highlighting the multifaceted involvement of SRP in mitigating Cd-induced AKI. Notably, pathways related to Cd-induced AKI that showed significant enrichment included the MAPK, apoptosis, and AKT signaling pathways. Previous studies have reported that the MAPK signaling pathway plays a key role in elevating Kim-1 levels in nephropathy and mediating the activation of macrophages [49], which is consistent with our results. Arg has also been reported to mitigate LPS-induced muscle injury via apoptosis and the AKT signaling pathway [50]. The Bupi Yishen Formula attenuates adenine-induced chronic kidney disease by inhibiting the AKT signaling pathway [51]. Our previous study also showed that peptides from oyster protein hydrolysates ameliorated Cd-induced hepatic injury by inhibiting oxidative damage and inflammatory responses [51]. These data suggest that these genes (*AKT1*, *CASP3*) and pathways (MAPK, inflammatory responses, and apoptosis) are potential targets and networks of SRP’s effects on Cd-induced AKI.

### 2.5. SRP Alleviated Serum Oxidative Indices in AKI Mice

**Figure 5:**
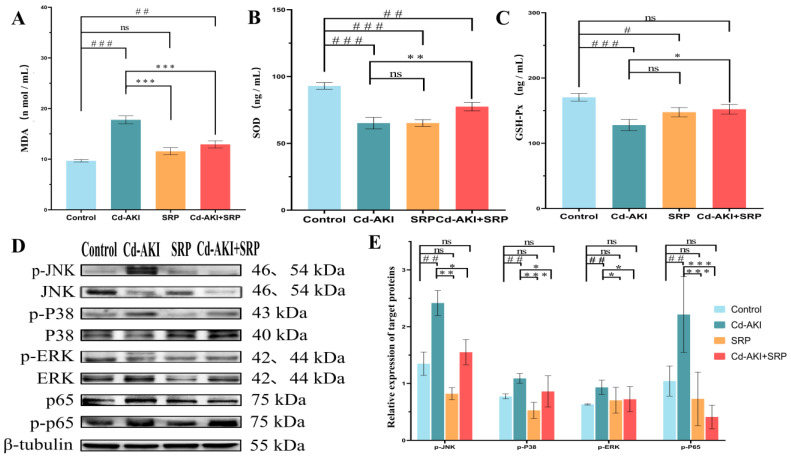
Effect of SRP on renal lipid oxidation. (**A**) MDA levels. (**B**) GSH-Px levels. (**C**) SOD levels. (**D**) Protein expression of the MAPK signaling pathway components in kidney tissue. (**E**) Protein expression of the MAPK signaling pathway components, quantified by densitometry and normalized to β-tubulin. All presented data represent the mean ± SD. Significant differences are indicated as follows: “^#^” indicates values that are significantly different from the control group (*p* < 0.05); “^##^” indicates values that are significantly different from the control group (*p* < 0.01); “^###^” indicates values that are significantly different from the control group (*p* < 0.001); “*” indicates values that are significantly different from the Cd-AKI group (*p* < 0.05); “**” indicates values that are significantly different from the Cd-AKI group (*p* < 0.01); “***” indicates values that are significantly different from the Cd-AKI group (*p* < 0.001); “ns” indicates no significant difference.

To investigate whether SRP alleviates Cd-induced AKI by regulating the MAPK pathway and its mediated oxidative stress response, we first analyzed serum MDA, GSH-Px, and SOD levels using biochemical kits (Figure 5A–C). Cd exposure considerably (*p* < 0.05) decreased the enzymatic activities of SOD and GSH-PX, while markedly (*p* < 0.05) increasing the levels of MDA compared to the control group. Co-administration of SRP and Cd resulted in a noticeable (*p* < 0.05) increase in the activities of SOD and GSH-PX enzymes, while significantly (*p* < 0.05) decreasing MDA levels compared to the Cd-treated group. The SRP-treated group demonstrated GSH-PX activities and SOD and MDA levels similar to those of the control mice (Figure 5). The results are consistent with those reported in [52]. In this study, we found that the kidney levels of MDA and SOD were significantly higher and lower, respectively, in mice after Cd administration compared with the levels detected in the blank control group, which indicated that Cd induced kidney damage. In contrast, the MDA and SOD levels in the groups treated with SRP were significantly lower and higher, respectively, than those in the Cd control group, indicating that SRP protected the mice against Cd-induced kidney damage and functioned as an antioxidant to protect kidney cells in this model.

Concurrently, we assessed renal tissue injury by WB. Previous studies have demonstrated an upregulation of the MAPK signaling pathway in Cd-induced AKI [53]. To validate the network pharmacology results and elucidate the mechanism of SRP, the expression and phosphorylation of MAPK pathway components (p38, ERK, and JNK) were measured. WB revealed that Cd induced the phosphorylation of p38, ERK, and JNK, whereas SRP inhibited their phosphorylation, particularly of p38 and p65 MAPK (Figure 5A,B). This result is in line with an earlier report in which the tetrapeptide SS-31, in a high-glucose-induced renal injury model, reduced oxidative stress and inhibited the activation of the p38 MAPK pathway [54]. Aquaporin-1 attenuates LPS-induced AKI by suppressing the p38 MAPK pathway [55]. The mechanism of action of SRP may be consistent with the protein peptide action pathway. Dietary Arg supplementation suppresses p65 signaling [56]. These data suggest a protective role of SRP through the MAPK signaling pathway.

### 2.6. SRP Reduced the Renal Inflammatory Reaction in AKI Mice

**Figure 6:**
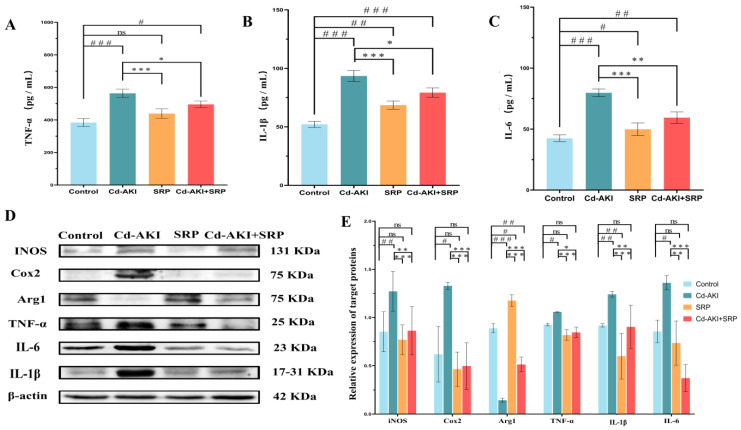
Effect of SRP on the renal inflammatory reaction. (**A**) TNF-α, (**B**) IL-1β, and (**C**) IL-6 levels. (**D**) Protein expression related to the inflammatory pathway in kidney tissue. (**E**) Protein expression of inflammatory pathway components, quantified by densitometry and normalized to β-actin. Significant differences are indicated as follows: “^#^” indicates that the values are significantly different from the control group (*p* < 0.05); “^##^” indicates that the values are significantly different from the control group (*p* < 0.001); “^###^” indicates that the values are significantly different from the control group (*p* < 0.01); “*” indicates that the values are significantly different from the Cd-AKI group (*p* < 0.05); “**” indicates that the values are significantly different from the Cd-AKI group (*p* < 0.01); “***” indicates that the values are significantly different from the Cd-AKI group (*p* < 0.001); “ns” indicates no significant difference.

To investigate whether SRP alleviated Cd-induced AKI by modulating the inflammatory response pathway, we analyzed serum TNF-α, IL-1β, and IL-6 levels using biochemical kits. The cytokines TNF-α, IL-1β, and IL-6 are recognized as triggers of inflammatory responses [57]. In this study, CdCl_2_ (2 mg/kg) treatment for 30 days significantly (*p* < 0.001) enhanced the levels of TNF-α, IL-1β, and IL-6 in the sera of the experimental mice (Figure 6). In contrast, SRP (50 mg/kg) significantly reduced the Cd-mediated enhancement of TNF-α (*p* < 0.05), IL-1β (*p* < 0.05), and IL-6 (*p* < 0.01) levels in the sera of mice (Figure 6).

Several previous studies have indicated that Cd exposure in kidney tissues can activate NF-κB, which increases IL-1, IL-6, and TNF-α levels, while decreasing IL-10 levels [58]. Conversely, SRP downregulation significantly reduced the IL-6, TNF-α, and IL-1β levels. These findings highlighted the potential therapeutic benefits of SRP in alleviating Cd-induced AKI by progressively modulating inflammatory responses.

Furthermore, we evaluated the expression of iNOS, Arg1, Cox2, TNF-α, and other inflammatory proteins (Figure 6D,E). Cd exposure significantly elevated inflammatory indices, including IL-1β, TNF-α, IL-6, NF-κB, iNOS, and Cox2 activity. In contrast, Cd notably decreased the expression of Arg1 in mouse kidney tissue. Notably, SRP administration significantly reduced inflammatory protein expression in injured kidney tissues (*p* < 0.05). These results are in line with earlier reports in which Arg support improved renal immune responses by reducing Cox2 levels [59], decreasing iNOS expression [60], and inducing Arg1 expression [61]. Previous studies have shown that heavy metals can directly increase the production of proinflammatory cytokines. Moreover, COX-2 and iNOS modulate the inflammatory response by producing prostaglandin E2 and nitric oxide, respectively. Nitric oxide can make the cell more vulnerable to ROS by reducing the intracellular glutathione content.

Recent studies have highlighted the significance of genetic factors in the pathogenesis of kidney disease. Al-Awaida et al. [62] evaluated the genetic association and expression of Notch-2/Jagged-1 in patients with type 2 diabetes mellitus and found significant alterations in these pathways, which are known to play crucial roles in cell differentiation, proliferation, and apoptosis. Incorporating findings from genetic studies such as this could provide a more comprehensive understanding of the molecular mechanisms underlying Cd-induced AKI and the therapeutic potential of SRP. Notch signaling, similar to the MAPK pathway targeted by SRP, may be involved in the regulation of inflammatory and apoptotic responses in kidney cells, suggesting potential synergistic pathways for therapeutic intervention.

### 2.7. SRP Suppressed the Renal Cell Apoptosis in AKI Mice

**Figure 7:**
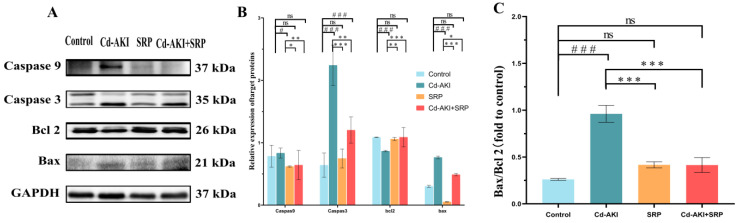
The effect of SRP on cell apoptosis in Cd-injured kidney tissues. (**A**) The expression of apoptotic proteins in kidney tissue. (**B**) Quantification by densitometry and normalized to GAPDH. (**C**) The relative expression ratio of apoptotic protein Bax and Bcl 2. All data are presented as the mean ± SD. “^#^” indicates values that significantly differ from the control group (*p* < 0.05). “^###^” indicates values that significantly differ the control group (*p* < 0.01). “*” indicates values that significantly differ from the Cd-AKI group (*p* < 0.05). “**” indicates values that significantly differ from the Cd-AKI group (*p* < 0.01). “***” indicates values that significantly differ from the Cd-AKI group (*p* < 0.001). “ns” indicates no significant difference.

A distinctive characteristic of Cd-induced AKI is apoptosis [63], and our KEGG enrichment analysis indicated that apoptosis may play a key role in alleviating Cd-induced AKI by SRP. Therefore, we examined the expression of apoptosis-related proteins. Cd exposure led to a significant (*p* < 0.001) decrease in the expression of the anti-apoptotic marker Bcl-2 while the expression of apoptotic markers (caspase-9, caspase-3, and Bax) was upregulated in SRP-treated mice when compared to the control group. However, co-treatment with Cd and SRP resulted in a significant (*p* < 0.05) reversal of the expression of these anti-apoptotic and apoptotic markers compared to the Cd-treated group. Administration of SRP alone resulted in the normal expression of these markers, which were comparable to those observed in control mice (Figure 7). Caspase-3, a vital apoptosis mediator, initiates the apoptotic cascade by activating other caspase enzymes [58]. Previous research has shown that exposure to Cd increases the expression of Bax and caspase-3 while decreasing Bcl-2 levels in renal tissue, thereby triggering apoptosis. The Bax/Bcl-2 ratio, which is commonly used to determine the level of apoptosis, was markedly reduced by SRP treatment (Figure 7B), confirming the protective role of SRP in apoptosis reduction (Figure 7C). These results are in line with earlier reports in which Arg ameliorated heavy metal-induced AKI by modulating the expression of renal markers of apoptosis (caspase-3, Bax, and Bcl-2) [64]. Pro alleviates AFB1-induced kidney injury by regulating apoptotic factors (Bax, Bcl-2, and cleaved caspase-3) [23]. The interplay between Bax and Bcl-2 is crucial for initiating the fundamental apoptotic pathway by facilitating the release of cytochrome c from mitochondria [58]. However, Arg support reduces mitochondrial dysfunction by increasing mitochondrial cytochrome c oxidase activity and reducing cytosolic cytochrome c activity [65,66]. Together, these data suggest that SRP effectively improves Cd-induced AKI by inhibiting Cd-triggered apoptosis via modulation of the expression of apoptotic factors (caspase-3, Bax, and Bcl-2) in the kidney.

Both oxidative and inflammatory pathways triggered by Cd may activate apoptosis, which plays a pivotal role in Cd-induced nephrotoxicity. Previous studies have reported that the occurrence of apoptosis involves p53 and its downstream targets and that the anti- and pro-apoptotic members of the Bcl-2 family are crucial effectors of p53-regulated apoptosis. In this study, we found that Cd exposure augmented apoptosis in the kidney. In mice, Bcl-2 expression was downregulated and Bax was upregulated after exposure to Cd, thus confirming the important role of Cd in triggering apoptosis. In this context, many natural substances have shown a promising role in positively modulating the apoptotic pathways after Cd treatment, such as selenium, Potentilla anserina polysaccharide, betulinic acid, myo-inositol, vitamin E, and quercetin [67]. In our study, we observed that SRP protected cells against Cd-induced apoptosis.

## 3. Materials and Methods

### 3.1. Chemicals

**Figure 8:**
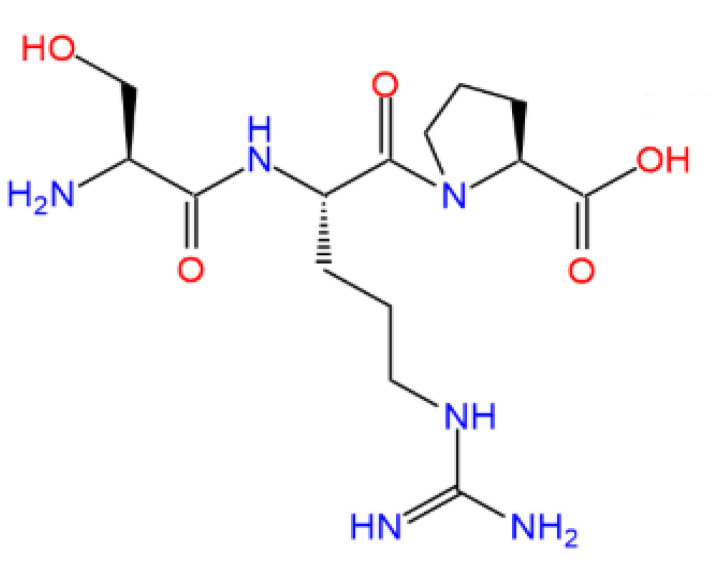
Chemical structure of SRP.

SRP was provided by Guangdong Medical University (purity: 98.5%, Figure 8).

### 3.2. Animal Housing and Acclimatization

C57BL/6 mice (male, 22–25 g) were procured from Zhuhai Bestest Bio-Test Co., Ltd. (Guangzhou, China) and housed in a temperature-controlled environment (20–22 °C) with ample food and water.

After one week of acclimatization to the breeding environment, 40 mice were randomly assigned to four groups (10 mice per group): (1) control, (2) SRP, (3) Cd-AKI, and (4) Cd-AKI + SRP. The control and SRP groups were injected intraperitoneally with 2 mg/kg saline, and the Cd-AKI and Cd-AKI + SRP groups were injected intraperitoneally with 2 mg/kg cadmium [68]. Saline (50 mg/kg) was administered orally to the control and Cd-AKI groups, and 50 mg/kg SRP was administered orally to the SRP and Cd-AKI + SRP groups after 1 h for 30 days [29]. Throughout the experiment, individual mouse weights were recorded every five days. All animal treatments and experimental protocols were approved by the Experimental Animal Ethics Committee of Guangdong Ocean University (approval number: GDOULAE-2023-031).

### 3.3. Biochemical Assessment of Blood Indices

Following the induction of Cd-induced AKI, blood samples were collected from the ocular region of the mice under ether anesthesia to minimize discomfort and pain. The collected blood samples were stored at 4 °C for 4–6 h and then centrifuged at 3000 rpm for 10 min under the same temperature conditions.

Serum creatinine (CRE) and blood urea nitrogen (BUN) levels were determined using specific kits from Nanjing Jianjieng Bioengineering Institute (C013-2-1, C011-2-1). Additionally, serum interleukin-1β (IL-1β), interleukin-6 (IL-6), tumor necrosis factor-alpha (TNF-α), superoxide dismutase (SOD), malondialdehyde (MDA), and glutathione peroxidase (GSH-Px) levels were quantified using kits obtained from Quanzhou Ruixin Biological Technology Co., Ltd., Quanzhou, China (RX203063M, RX203049M, RX202412M, RX201865M, RXJ203032M, RX201373M).

### 3.4. Renal Histopathological Assessment

Renal histopathological assessments were performed as previously described [69]. Kidney specimens were meticulously collected and promptly fixed in a 4% paraformaldehyde solution. The specimens were embedded in paraffin for further processing. Tissue sections with a thickness of 4 μm were prepared from the paraffin-embedded kidney samples. Following deparaffinization and rehydration, the kidney sections were stained with hematoxylin and eosin (HE) and Masson’s trichrome (MS), as previously described [70].

### 3.5. Immunohistochemical Analysis

Immunohistochemical detection was performed to assess the presence of Kim-1 as previously described [71]. Previously prepared kidney sections were deparaffinized and rehydrated. The sections were treated with 0.3% H_2_O_2_ for 10 min, followed by antigen retrieval using 10% citrate buffer (P0081, Beyotime). Subsequently, the sections were incubated overnight with primary antibodies at 4 °C. After washing with PBS, the sections were incubated with a secondary antibody for 30 min and treated with DAB (Vector Laboratories). Finally, hematoxylin was used for counterstaining, and images were acquired using a microscope (Nikon, ECLIPSE, 50i, Shanghai, China) and analyzed using the ImageJ V1.8.0 software. The primary antibodies and dilutions used were as follows: Kim-1 (1:100; AF1817-SP, R&D Systems, Minneapolis, MN, USA).

### 3.6. Network Pharmacology Analysis

#### 3.6.1. In Silico ADMET and Drug-Likeness Prediction

Considering SRP as a potential drug candidate, various critical parameters, including physicochemical properties, drug likeness, lipophilicity, solubility, and other pharmacokinetic profiles, were predicted computationally (Table S1). Swiss ADME, a web server-based prediction tool developed and maintained by the Swiss Institute of Bioinformatics (SIB), was utilized to predict and collect information on the absorption, distribution, metabolism, excretion (ADME), and pharmacokinetic properties [72]. The OSIRIS Property Explorer, another standalone open-source tool, was used to theoretically predict the toxicity risk of SRP, grading the predicted toxicity risk as high, medium, or low toxicity risk factors.

#### 3.6.2. Target Prediction Using Network Pharmacology

The SuperPred platform (prediction.charite.de), PharmMapper (PharmMapper (lilab-ecust.cn)), and Swiss Target Prediction (SwissTargetPrediction) were employed to predict potential genes/targets associated with SRP. This process involved converting protein names to gene names by referencing the UniProt database (UniProt).

Simultaneously, a disease target library for Cd-induced AKI was established by integrating data from CTD (The Comparative Toxicogenomics Database; ctdbase.org; accessed on 25 January 2023), DisGeNET (a database of gene-disease associations; accessed on 25 January 2023), NCBI’s National Center for Biotechnology Information (https://www.ncbi.nlm.nih.gov/ accessed on 25 January 2023); GeneCard (GeneCards—Human Genes|Gene Database|Gene Search);accessed on 25 January 2023), and OMIM. The intersection of drug targets and disease targets was determined using a Venny2.1.0 analysis as previously described [73].

Subsequently, a comprehensive network model was created using Cytoscape 3.7. To further elucidate potential therapeutic mechanisms, the signaling pathways of common targets were analyzed using DAVID (DAVID Functional Annotation Bioinformatics Microarray Analysis (ncifcrf.gov); accessed on 28 January 2023), with a focus on Gene Ontology (GO) and the Kyoto Encyclopedia of Genes and Genomes (KEGG) analyses. This comprehensive analysis aimed to identify the key active ingredients, signaling pathways, and important targets for the treatment of Cd-induced AKI.

### 3.7. Western Blot Analysis

Western blot analysis was performed as previously described [74]. Total protein from renal tissue was isolated using RIPA lysis buffer (P0013B, Beyotime, Shanghai, China). The total protein concentration was measured using a BCA protein detection kit (P0010, Beyotime, Shanghai, China). A polyacrylamide SDS-PAGE gel (6–15%) (P0012A, Beyotime, Shanghai, China) was used to separate the target protein using a vertical electrophoresis system. The isolated target protein was analyzed by immunoblotting and detected using an enhanced chemiluminescence kit (P0018M, Beyotime, Shanghai, China). The protein bands were accurately captured using a multifunctional ultrasensitive imaging system (SHST, Shanghai, China). The ImageJ software was used to standardize the relative expression levels of each protein during signal development.

### 3.8. Statistical Analysis

All experiments were conducted in triplicate, unless otherwise specified. Data are presented as mean ± SD, and analyses were performed using SPSS software (version 16.0; SPSS Inc., Chicago, IL, USA). One-way analysis of variance (ANOVA) followed by the Student–Newman–Keuls test was used for comparisons. Statistical significance was set at *p* < 0.05.

## 4. Conclusions

**Figure 9:**
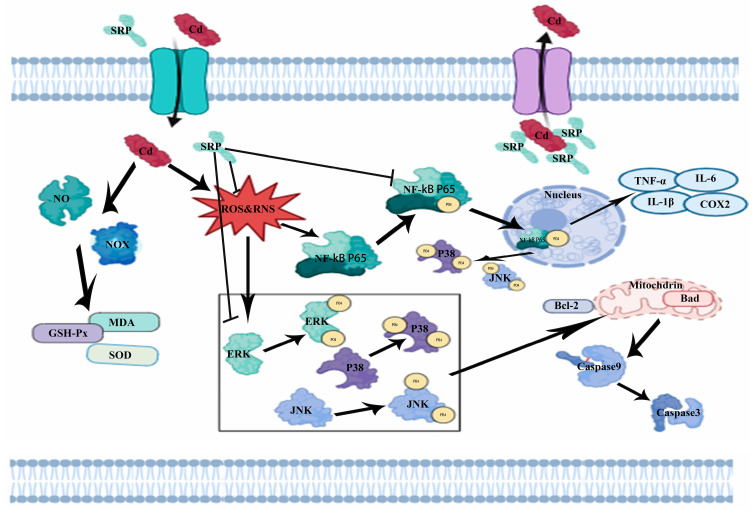
Schematic overview of the probable protective mechanism of SRP against Cd-induced AKI. The black arrows (→) indicate downstream cellular events, and the flat arrows (T) indicate pathological events limited by SRP.

In summary, the present study demonstrated the ability of SRP to attenuate Cd-induced renal injury in mice. The nephroprotective effects of SRP against Cd-mediated nephrotoxicity may be attributed to its anti-inflammatory, anti-apoptotic, and antioxidant nature (Figure 9). Hence, SRP may be considered a valuable adjunct agent for the management of Cd toxicity in vivo. However, the limitation of this study is that it was conducted on animal models, and it is necessary to conduct clinical trials in the future to determine the effectiveness and safety of SRP in humans.

## Data Availability

The data presented in this study are available on request from the corresponding author.

## Supplementary Materials

The following supporting information can be downloaded at: https://www.mdpi.com/article/10.3390/md22060286/s1, Table S1: In silico prediction of drug-likeness and ADMET profiles of SRP.
