# Supplementary material for: A Tripeptide (Ser-Arg-Pro, SRP) from Sipunculus nudus L. Improves Cadmium-Induced Acute Kidney Injury by Targeting the MAPK, Inflammatory, and Apoptosis Pathways in Mice
Source: Mar Drugs. 2024 Jun 20;22(6):286. doi: 10.3390/md22060286 (PMC11204732; doi:10.3390/md22060286)
Supplement: Supplementary file 1 [file marinedrugs-22-00286-s001.zip › marinedrugs-3028390-supplementary.pdf]

Supporting Information

A Tripeptide (Ser-Arg-Pro, SRP) from *Sipunculus nudus* L Improves Cadmium-Induced Acute Kidney Injury by Targeting the MAPK, Inflammatory and Apoptosis Pathways in Mice

Yanmei Pan ‡[a], Zhilan Peng ‡[b], Zhijia Fang*[a], Iddrisu Lukman [a], Lijun Sun[a], Qi Deng[a], Ravi Gooneratne[c]

[a] College of Food Science and Technology, Guangdong Provincial Key Laboratory of Aquatic Product Processing and Safety, Guangdong Provincial Engineering Technology Research Center of Marine Food, Key Laboratory of Advanced Processing of Aquatic Products of Guangdong Higher Education Institution, Guangdong Ocean University, Zhanjiang 524088, China

[b] The Marine Biomedical Research Institute of Guangdong Zhanjiang, Zhanjiang 524023, China

[c] Department of Wine, Food and Molecular Biosciences, Lincoln University, Lincoln, Canterbury 7647, New Zealand

‡ These authors contributed equally to this work.

*Corresponding Author: fangzj@gdou.edu.cn (Z. Fang).

Table of contents

Supplementary table page 2

Supplementary table

Table S1. In silico prediction of drug-likeness and ADMET profiles of SRP.

Properties	Profile	Values
Properties under Lipinski's rule of five	Molecular weight	358.40
	Hydrogen bond acceptor	7
	Hydrogen bond donor	7
	ALog	-2.87
Other physico-chemical properties	Number of rotatable bonds	12
	Molecular refractivity	92.05
	Topological polar surface area	194.86
Water solubility	Log *S*	Soluble
Pharmacokinetics profiles	GI absorption	Low
	log *K*p (cm/s)	−12.81
	CYP1A2 inhibitor	No
	CYP2C19 inhibitor	No
	CYP2C9 inhibitor	No
	CYP2D6 inhibitor	No
	CYP3A4 inhibitor	No
Drug-likeness profiles	Bioavailability score	0.5714
Toxicity risk assessment	Mutagenicity risk	Low
	Irritating effect	Low
	Reproductive toxicity effect	Low

Properties	Profile	Values
	Tumorigenicity risk	Low
